# A Deep Learning Framework Predicts Rectal Cancer Treatment Response via Pretreatment 3D Transrectal Ultrasound: A Multicenter Study

**DOI:** 10.1002/mco2.70982

**Published:** 2026-09-09

**Authors:** Min Liu, Yiwen Yu, Xuebin Zou, Ying Liao, Lichao Mou, Xing Zhao, Juan Fu, Lina Tang, Xiaomao Luo, Guangjian Liu, Fang Li, Jingliang Hu, Anhua Li, Jianwei Wang, Ruohan Guo, Jianhua Zhou

**Affiliations:** ^1^ Department of Ultrasound State Key Laboratory of Oncology in South China Sun Yat‐Sen University Cancer Center Guangdong Provincial Clinical Research Center for Cancer Guangzhou China; ^2^ MedAI Technology (Wuxi) Co., Ltd Wuxi China; ^3^ Department of Diagnostic Ultrasound Fujian Cancer Hospital and Fujian Medical University Cancer Hospital Fuzhou China; ^4^ Department of Medical Ultrasound Yunnan Cancer Hospital & The Third Affiliated Hospital of Kunming Medical University Kunming China; ^5^ Department of Ultrasound The Sixth Affiliated Hospital Sun Yat‐Sen University Guangzhou China; ^6^ Department of Ultrasound School of Medicine Chongqing University Cancer Hospital Chongqing University Chongqing China

**Keywords:** 3D transrectal ultrasound, automatic segmentation, deep learning, neoadjuvant therapy, pathological complete response, rectal cancer

## Abstract

Accurate prediction of pathological complete response (pCR) following neoadjuvant therapy is crucial for personalized treatment planning in patients with locally advanced rectal cancer (LARC). This study aimed to explore the application of 3D transrectal ultrasound (TRUS) for this purpose. In this study, 538 LARC patients from five hospitals were enrolled and divided into training (*n* = 348), internal validation (*n* = 87), and external validation (*n* = 103) cohorts. Using pretreatment 3D TRUS data of the rectal tumors, a deep learning framework comprising an automated segmentation model and a pCR prediction model was constructed and validated. The segmentation model achieved an excellent Dice score of 0.89. The predictive model, when trained on data sampled at 9° or 18° intervals, yielded area under the curve (AUC) values of 0.92 (internal validation) and 0.85–0.86 (external validation), with accuracies of 86.2%–90.8% and 81.6%–83.5%, respectively; decision‐curve analysis also demonstrated clinically meaningful net benefits. This study presents the first deep learning framework based on 3D TRUS for pCR prediction in LARC patients, offering an automated and reproducible tool that may support clinical decision‐making in rectal cancer management. However, further validation in larger, multi‐device cohorts is essential before clinical application.

## Graphical abstract

Not all rectal cancer patients achieve pathological complete response (pCR) after neoadjuvant therapy, and the predictive potential of 3D transrectal ultrasound (TRUS) in this setting remains unexplored (A). Multi‐center pre‐treatment data were collected (B) and used to develop a semi‐supervised segmentation model and a 3D‐ResNet predictive model (C). The integrated framework demonstrated robust pCR predictive performance across independent cohorts (D) and may aid in clinical decision‐making.

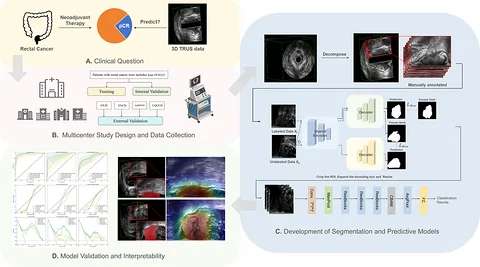

## 1 Introduction

Rectal cancer is a leading global malignancy, with over 700,000 new cases diagnosed annually [1, 2]. A substantial proportion of cases are classified as locally advanced rectal cancer (LARC), defined by T3‐4 tumor invasion or lymph node involvement. Current guidelines recommend neoadjuvant treatment followed by total mesorectal excision surgery. However, due to the biological heterogeneity of the disease, treatment responses in LARC are highly variable [3, 4]. Meanwhile, there is growing interest in non‐operative, organ‐preserving “watch‐and‐wait” strategies, in which selected patients achieving potential pathological complete response (pCR) have shown favorable 5‐year survival outcomes without surgery‐related morbidity or functional complications [5, 6, 7]. This underscores the critical need for accurate prediction of pCR in rectal cancer. Safe expansion of this strategy depends heavily on the development of robust tools for patient selection.

Artificial intelligence (AI) offers promising approaches to quantitative medical image analysis [8]. Several studies have reported promising predictive performances of AI models using pretreatment images to distinguish pCR and non‐pCR in rectal cancer patients [9, 10, 11, 12, 13]. This indicates the capacity of AI to extract prognostic information relevant to neoadjuvant outcomes from medical images obtained at diagnosis. However, previous studies predominantly adopted MRI data and relied heavily on manual segmentation. Clinical application of these AI models is restricted not only by MRI's relatively high cost, long acquisition time, and limited availability but also by the labor‐intensive and subjective nature of manual segmentation, which introduces potential observer variability and poses a huge barrier to building large‐scale datasets. It is imperative to develop novel models to address these limitations.

Transrectal ultrasound (TRUS) offers a more accessible and cost‐effective imaging alternative that allows real‐time evaluation of tumors [14, 15]. Compared to conventional 2D TRUS, 3D TRUS has the advantages of a broader field of view capturing complex spatial patterns and operator‐independent nature [16]. Thus, it holds strong potential to facilitate AI integration into diverse clinical settings. In previous studies, convolutional neural network (CNN)‐based models using 3D ultrasound data have shown promising performance in tumor segmentation and outcome prediction, yet their applications remain largely confined to thyroid and breast cancers [17, 18, 19]. To the best of our knowledge, no prior studies have either developed segmentation tools specifically for rectal cancer on 3D TRUS images, or explored the use of these data for pCR prediction in rectal cancer.

To address this gap, this study aimed to develop and validate a novel deep learning framework leveraging pretreatment 3D TRUS data for pCR prediction in LARC. This framework incorporated a semi‐supervised segmentation model that closely aligns with manual annotations and a robust 3D‐ResNet‐based predictive model demonstrating reliable performance across multicenter datasets, thereby offering a promising tool to aid in personalized treatment planning.

## 2 Results

### 2.1 Patient Characteristics

**FIGURE 1:**
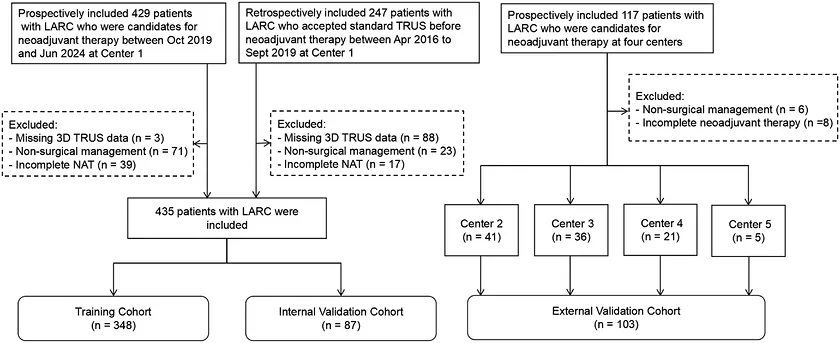
Flowchart of patient enrollment and allocation. LARC = locally advanced rectal cancer; TRUS = transrectal ultrasound; NAT = neoadjuvant therapy.

The patient selection process and dataset construction are depicted in Figure 1. Ultimately, 538 rectal cancer patients were included and divided into training (*n* = 348), internal validation (*n* = 87), and external validation cohorts (*n* = 103).

The baseline clinicopathological characteristics of patients in each cohort are summarized and compared in Table 1. The age was comparable across groups (training: 56.8 ± 10.4 years; internal validation: 57.2 ± 10.4 years; external validation: 56.0 ± 11.7 years; *p = *0.71), and the sex distribution was balanced (male: 58.9% [205/348], 63.4% [56/87], and 59.2% [61/103], respectively; *p = *0.64). CEA and CA199 levels did not significantly differ (*p = *0.44; *p = *0.31). The overall pCR rate was 27.1% (146/538) and showed no significant difference across groups (training: 27.0% [94/348]; internal validation: 27.6% [24/87]; external validation: 27.2% [28/103]; *p* = 0.31). Regarding tumor‐related characteristics, the study populations exhibited comparable tumor sizes (*p = *0.36), pretreatment T stage distribution (*p = *0.21), and nodal involvement (*p = *0.083). Overall, the three cohorts were well balanced for most clinicopathological characteristics; the only observed inter‐group difference was in tumor differentiation (*p = *0.037).

**TABLE 1:** Baseline clinicopathological characteristics of patients.

Characteristic	Training (*n* = 348)	Internal validation (*n* = 87)	External validation (*n* = 103)	*p* value
Age (years)^a^	56.8 ± 10.4	57.2 ± 10.4	56.0 ± 11.7	0.71
Sex				0.64
Female	143 (41.1%)	31 (35.6%)	42 (40.8%)	
Male	205 (58.9%)	56 (63.4%)	61 (59.2%)	
CEA (ng/mL)				0.44
> 5	153 (44.0%)	35 (40.2%)	47 (45.6%)	
≤ 5	195 (56.0%)	52 (59.8%)	48 (46.6%)	
N/A	0 (0.0%)	0 (0.0%)	8 (7.8%)	
CA199 (U/mL)				0.31
> 35	51 (14.7%)	11 (12.6%)	19 (18.4%)	
≤ 35	297 (85.3%)	76 (87.4%)	75 (72.8%)	
N/A	0 (0.0%)	0 (0.0%)	9 (8.7%)	
Tumor size (cm)^a^	4.1 ± 1.3	4.0 ± 1.2	4.0 ± 1.0	0.36
Pretreatment T stage				0.21
1	2 (0.6%)	0 (0.0%)	0 (0.0)	
2	8 (2.3%)	6 (6.9%)	7 (6.8%)	
3	245 (70.4%)	57 (65.5%)	66 (64.1%)	
4	93 (26.7%)	24 (27.6%)	30 (29.1%)	
Pretreatment N stage				0.083
0	24 (6.9%)	9 (10.3%)	13 (12.6%)	
+	324 (93.1%)	78 (89.7%)	90 (87.4%)	
TRG^b^				0.31
0 (pCR)	94 (27.0%)	24 (27.6%)	28 (27.2%)	
1	76 (21.8%)	20 (23.0%)	22 (21.4%)	
2	148 (42.5%)	31 (35.6%)	49 (47.6%)	
3	31 (8.6%)	12 (13.8%)	4 (3.9%)	
Tumor differentiation				0.037
M/D or W/D	271 (77.9%)	72 (82.8%)	89 (86.4%)	
P/D or P‐M/D	62 (17.8%)	9 (10.3%)	7 (6.8%)	
NA	15 (4.3%)	6 (6.9%)	7 (6.8%)	

Abbreviations: CA199, carbohydrate antigen 19‐9; CEA, carcinoembryonic antigen; M/D, moderately differentiated; NA, not applicable; P/D, poorly differentiated; pCR, pathological complete response P‐M/D, poorly to moderately differentiated; TRG, tumor regression grade; W/D, well differentiated.

^a^ Data are means ± standard deviations.

^b^ Tumor regression grading (TRG) was evaluated as described in the American Joint Committee on Cancer (AJCC) and College of American Pathologists (CAP) TRG system. See Table S6 for details.

*p* values were calculated using the independent *t*‐test or the Mann–Whitney *U* test for continuous variables, and chi‐squared test for categorical variables.

**FIGURE 2:**
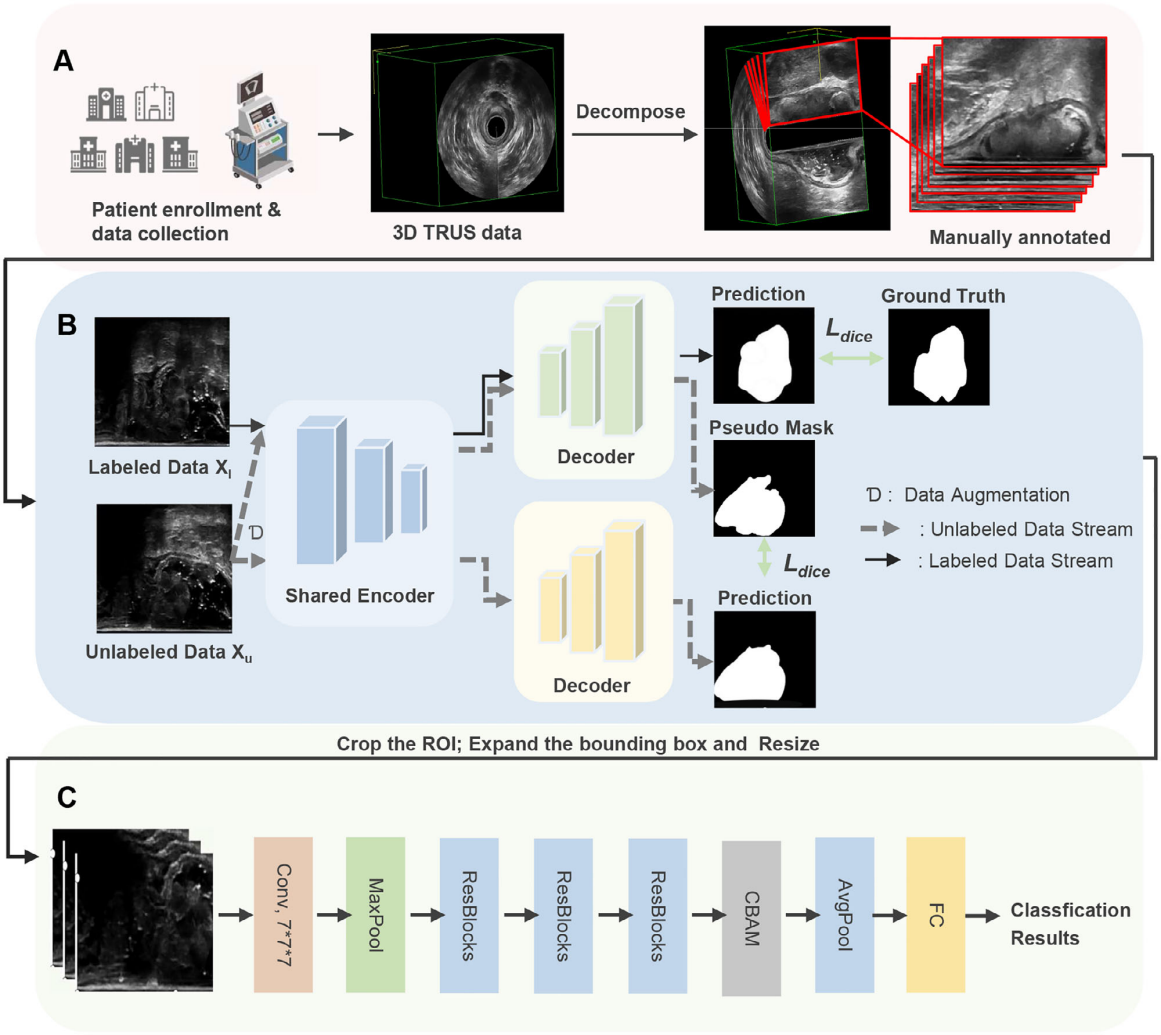
Workflow of this study and schematic diagram of the model architecture. (A) The process of patient enrollment and data collection. (B) The development of semi‐supervised learning segmentation model. (C) The development of the 3D‐ResNet‐based predictive model for pathological complete response incorporating a convolutional block attention module (CBAM). CBAM = convolutional block attention module; FC = fully connected; ROI = region of interest; TRUS = transrectal ultrasound.

Figure 2 shows the workflow of this study and the overall architecture of the predictive framework, which comprises a semi‐supervised model for rectal tumor segmentation and a 3D‐ResNet model for pCR prediction.

### 2.2 Segmentation Accuracy and Subgroup Stability of the Semi‐Supervised Model

**FIGURE 3:**
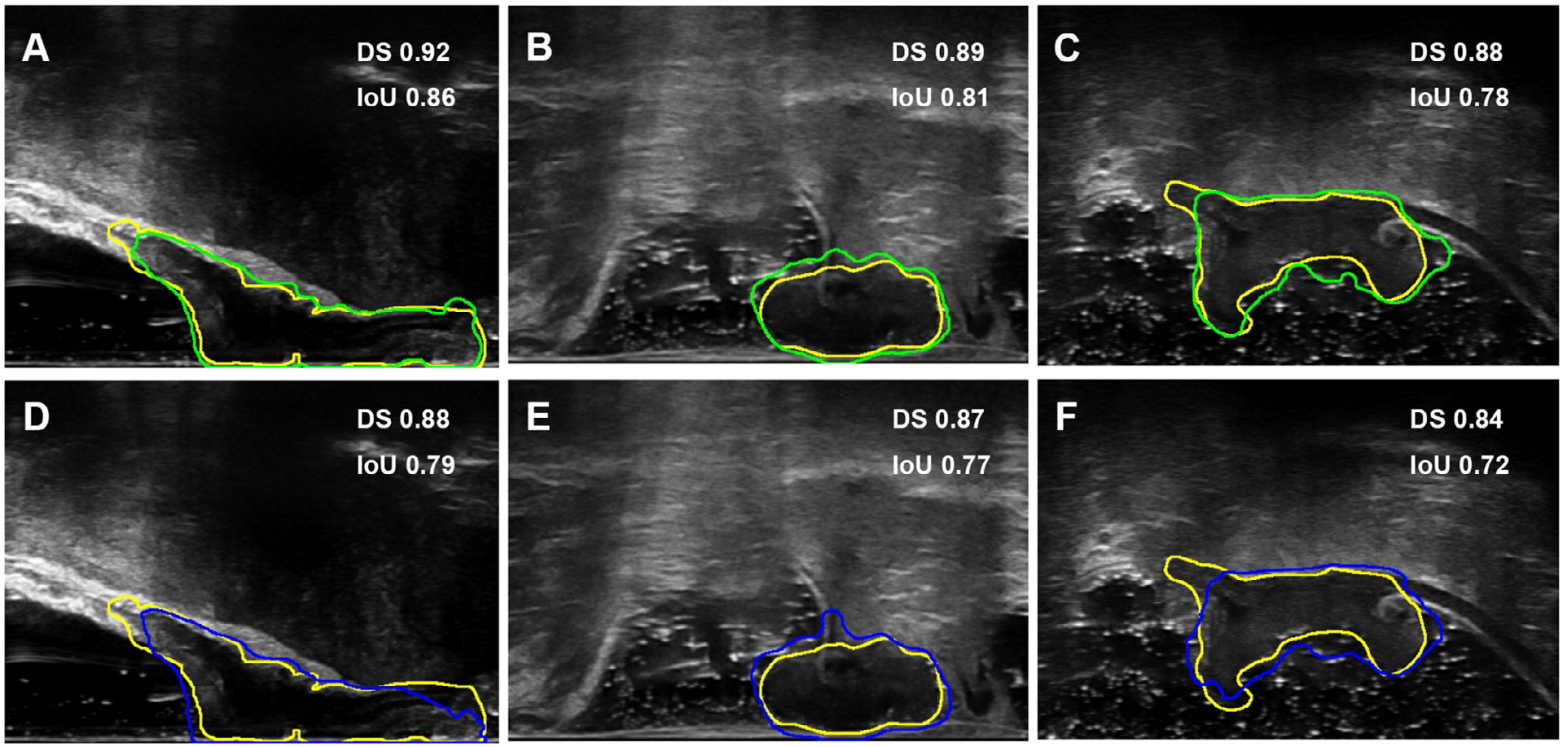
Examples of tumor segmentation by different models. (A, D, B, E, C, and F) Frames extracted from 3D transrectal ultrasound data showing different rectal lesions from three patients, respectively, comparing the regions of interest (ROIs) depicted by the standard U‐Net model (blue line), the semi‐supervised learning segmentation model (green line), and an experienced radiologist (yellow line). Dice scores and IoUs were calculated for each pair of ROIs. DS = Dice score; IoU = intersection over union.

Using 590 frames extracted from 3D TRUS data of 29 rectal lesions, the segmentation accuracy of the semi‐supervised model and the standard U‐Net model against manual annotations was evaluated. Overall, the semi‐supervised model achieved a Dice score of 0.89 and an intersection over union (IoU) of 0.82, significantly superior to the standard U‐Net model (Dice score 0.86, IoU 0.77; *p* < 0.001). When stratified by pretreatment T stage, tumor size, and distance from the anal verge, the semi‐supervised model consistently outperformed the standard U‐Net across all subgroups (Table S1, all *p *< 0.001). Figure 3 provides a visual comparison of representative tumor segmentations, illustrating that the semi‐supervised model produced ROI contours that matched the expert manual annotations more closely than those generated by the standard U‐Net model.

Table 2 further presents the segmentation performance of the semi‐supervised model across different tumor subgroups. Segmentation performance, as measured by the Dice score and IoU, showed no significant differences (*p* > 0.05) by tumor size (≥ 4.0 cm vs. < 4.0 cm) or by distance from the anal verge (≥ 5 cm vs. < 5 cm). However, performance was significantly higher for T3 lesions compared with T1‐2 lesions (Dice: 0.90 vs. 0.86, *p* = 0.032; IoU: 0.82 vs. 0.76, *p* = 0.013) and T4 lesions compared with T1‐2 lesions (IoU: 0.82 vs. 0.76, *p* = 0.018). In general, the semi‐supervised model exhibited robust segmentation capability across rectal tumors with diverse clinicopathological characteristics.

**TABLE 2:** Distribution of Dice scores and IoUs of semi‐supervised segmentation model across different subgroups.

Characteristic	Dice score	*p* value	IoU	*p* value
**Pretreatment T stage**				
T1‐2	0.86 ± 0.13		0.76 ± 0.15	
T3	0.90 ± 0.08	0.032^a^	0.82 ± 0.10	0.013^a^
T4	0.90 ± 0.11	0.065^a^	0.82 ± 0.14	0.018^a^
**Tumor size**		0.57		0.29
< 4.0 cm	0.89 ± 0.08		0.81 ± 0.12	
≥ 4.0 cm	0.90 ± 0.10		0.82 ± 0.12	
**Distance from anal verge**		0.052		0.12
< 5.0 cm	0.90 ± 0.06		0.83 ± 0.09	
≥ 5.0 cm	0.89 ± 0.11		0.81 ± 0.14	

Abbreviation: IoU = intersection over union.

^a^ Compared to T1‐2 lesions.

Data are means ± standard deviations. *p* values were calculated using the independent *t*‐test for continuous variables.

### 2.3 Predictive Performance of Models by Sampling Intervals and Differentiation Grade

**FIGURE 4:**
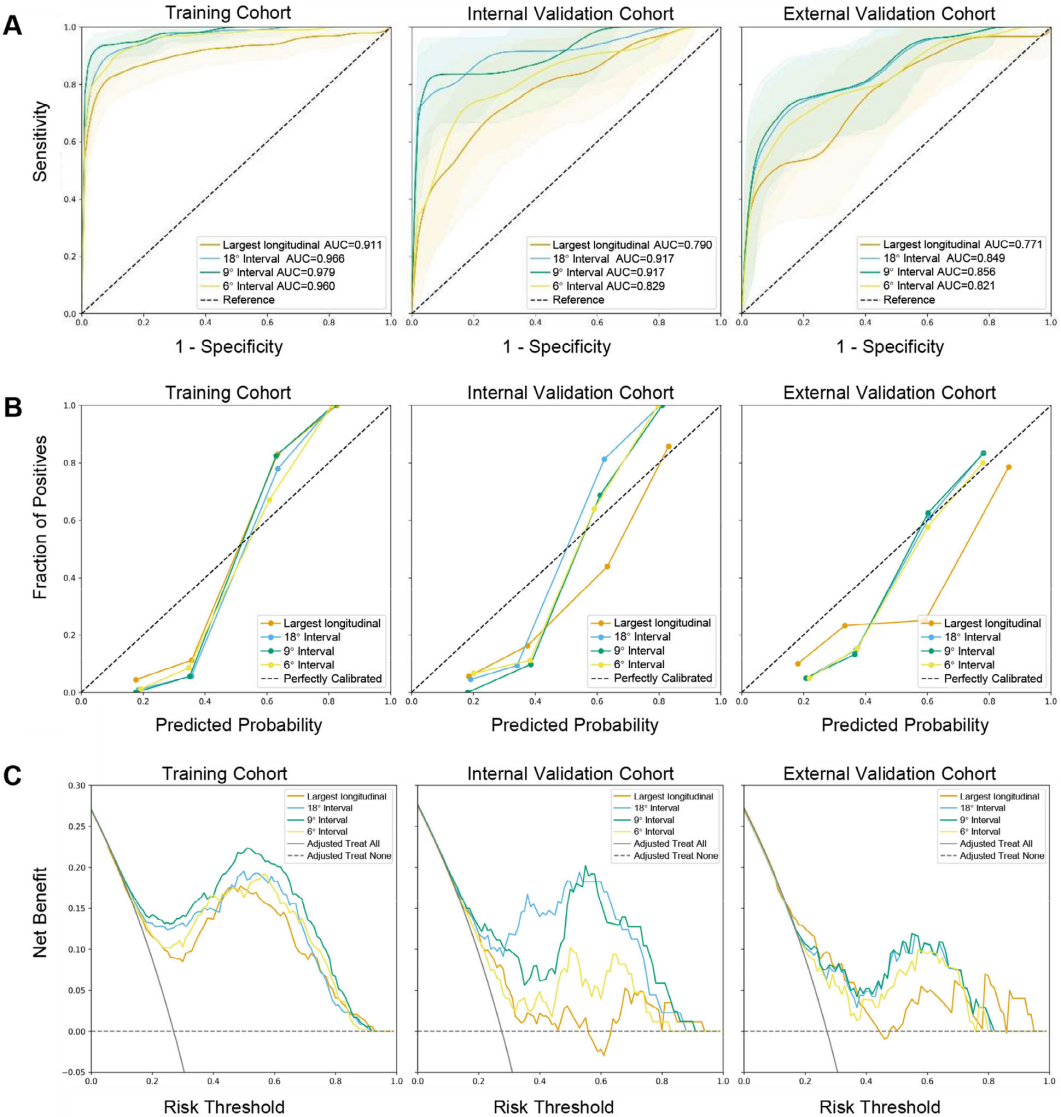
Performance of the deep learning models for pCR prediction in rectal cancer. (A) ROC curves of the predictive models with different sampling intervals in the training, internal validation, and external validation cohorts. **(B)** Calibration curves of the predictive models with different sampling intervals in the three cohorts. The curves show different degrees of agreement between the model‐predicted and observed pCR probabilities with different sampling intervals. **(C)** DCA curves of the predictive models with different sampling intervals in the three cohorts. The plot shows the net benefit across a range of risk thresholds of the models compared with intervention in all participants (all) or no intervention (none). AUC = area under the curve; DCA = decision curve analysis; pCR = pathological complete response; ROC = receiver operating characteristic.

The 3D‐ResNet predictive models developed from various sampling intervals of 3D TRUS data for predicting pCR in LARC patients were evaluated across the cohorts (Table 3 and Figure 4). In the training cohort, all models achieved excellent AUCs ranging from 0.91 to 0.98. Corresponding accuracy varied between 89.7% and 94.3%. The 9°‐interval model demonstrated the best predictive efficiency, achieving AUCs of 0.92 (95% CI, 0.83–0.98) and 0.86 (95% CI, 0.77–0.94) in the internal and external validation cohorts, with corresponding accuracies of 86.2% and 83.5% at the optimal cut‐off of 0.485. The performance of the 18°‐interval model was comparable at the optimal cut‐off of 0.500, producing AUCs of 0.92 (95% CI, 0.83–0.98) and 0.85 (95% CI, 0.76–0.93), and attaining accuracies of 90.8% and 81.6% in the respective cohorts. The calibration performance of the models with different sampling intervals was assessed across multiple cohorts, demonstrating good agreement between predicted probabilities and actual outcomes (*p > *0.05) (Figure 4B). Furthermore, decision curve analysis indicated that the 9°‐ and 18°‐interval model provided relatively good benefit for predicting pCR in all cohorts (Figure 4C).

**TABLE 3:** The performance of predictive models using different sampling intervals.

	AUC (95% CI)	Accuracy^a^	Sensitivity^a^	Specificity^a^	PPV^a^	NPV^a^
**Training cohort**						
Largest longitudinal	0.91 (0.86–0.95)	89.7% (312/348) [86.0%–92.5%]	81.9% (77/94) [73.3%–88.3%]	92.5% (235/254) [88.6%–95.2%]	80.2% (77/96) [71.7%–86.7%]	93.3% (235/252) [89.5%–95.8%]
18°‐interval	0.97 (0.94–0.98)	92.5% (322/348) [89.3%–94.9%]	90.4% (85/94) [82.6% – 95.1%]	93.3% (237/254) [89.5% – 95.9%]	83.3% (85/102) [74.8%–89.4%]	96.3% (237/246) [93.1%–98.2%]
9°‐interval	0.98 (0.96–0.99)	94.3% (328/348) [91.3%–96.3%]	91.5% (86/94) [83.9%–95.8%]	95.3% (242/254) [91.9%–97.4%]	87.8% (86/98) [79.8%–93.0%]	96.8% (242/250) [93.4%–98.5%]
6°‐interval	0.96 (0.93–0.98)	89.7% (312/348) [86.0%–92.5%]	91.5% (86/94) [83.9%–95.8%]	89.0% (226/254) [84.5%–92.5%]	75.4% (86/114) [66.7%–82.6%]	96.6% (226/234) [93.5%–98.4%]
**Internal validation cohort**						
Largest longitudinal	0.79 (0.66–0.90)	69.0% (60/87) [58.6%–77.7%]	75.0% (18/24) [54.8% – 88.3%]	66.7% (42/63) [54.3% – 77.1%]	46.2% (18/39) [31.5% – 61.4%]	87.5% (42/48) [75.0% – 94.5%]
18°‐interval	0.92 (0.83–0.98)	90.8% (79/87) [82.7%–95.5%]	79.2% (19/24) [59.1%–91.2%]	95.2% (60/63) [86.4%–98.9%]	86.4% (19/22) [65.8%–96.1%]	92.3% (60/65) [82.8%–97.1%]
9°‐interval	0.92 (0.83–0.98)	86.2% (75/87) [77.3%–92.1%]	83.3% (20/24) [63.5%–93.9%]	87.3% (55/63) [76.6%–93.7%]	71.4% (20/28) [52.8%–84.9%]	93.2% (55/59) [83.4%–97.8%]
6°‐interval	0.83 (0.72–0.92)	78.2% (68/87) [68.4%–85.6%]	75.0% (18/24) [54.8%–88.3%]	79.4% (50/63) [67.9%–87.5%]	58.1% (18/31) [40.8%–73.6%]	89.3% (50/56) [78.5%–95.0%]
**External validation cohort**						
Largest longitudinal	0.77 (0.66–0.87)	68.9% (71/103) [59.5%–77.1%]	53.6% (15/28) [35.8%–70.5%]	74.7% (56/75) [63.8%–83.1%]	44.1% (15/34) [28.9%–60.5%]	81.2% (57/69) [70.4%–88.7%]
18°‐interval	0.85 (0.76–0.93)	81.6% (84/103) [73.1%–87.9%]	67.9% (19/28) [49.4%–82.1%]	86.7% (65/75) [77.2%–92.6%]	65.5% (19/29) [47.5%–80.1%]	87.8% (65/74) [78.8%–93.4%]
9°‐interval	0.86 (0.77–0.94)	83.5% (86/103) [75.3%–89.5%]	75.0% (21/28) [56.6%–87.5%]	86.7% (65/75) [77.2%–92.6%]	67.7% (21/31) [50.4%–81.4%]	90.3% (65/72) [81.6%–95.3%]
6°‐interval	0.82 (0.73–0.91)	75.7% (78/103) [66.9%–82.9%]	75.0% (21/28) [56.6%–87.5%]	76.0% (57/75) [65.2%–84.3%]	53.8% (21/39) [38.6%–68.4%]	89.1% (57/64) [79.2%–94.7%]

Abbreviations: AUC = areas under the receiver operating characteristic curve; CI = confidence interval; NPV = negative predictive value; PPV = positive predictive value.

^a^ Values are presented as point estimate % (numerator/denominator) [95% confidence interval].

The differences of AUCs were further compared between models with different sampling intervals in the internal and the external cohorts (Table 4 and Figure S1). In the internal validation cohort, both the 18°‐ and the 9°‐interval models yielded excellent AUCs of 0.92, superior to the model using the largest longitudinal frames (AUC 0.79) with borderline statistical significance (*p = *0.084 and *p = *0.092, respectively) and △AUCs of 0.128 and 0.124, respectively. In the external validation cohort, the 18°‐ and the 9°‐interval models showed AUCs of 0.86 and 0.85, respectively, significantly higher than those of the model using the largest longitudinal frames and the 6°‐interval model (AUCs 0.77 and 0.82, respectively; both *p* < 0.05), and △AUCs of 0.080–0.084 and 0.029–0.034, respectively. Overall, the 9°‐ and the 18°‐interval models demonstrated superior predictive performance over the 6°‐interval and the single‐frame models in external validation, with favorable trends also observed in internal validation.

**TABLE 4:** Comparison of AUCs of the predictive models with different sampling interval.

	Compare to model based on largest longitudinal frame		Compare to 6°‐interval model	
	△AUC (95% CI)	*p* value	△AUC (95% CI)	*p* value
**Internal validation**				
Largest longitudinal	—	—	−0.041 (−0.110, 0.024)	0.24
18°‐interval	0.128 (−0.018, 0.271)	0.084	0.088 (−0.056, 0.216)	0.23
9°‐interval	0.124 (−0.027, 0.270)	0.092	0.084 (−0.062, 0.219)	0.26
6°‐interval	0.041 (−0.024, 0.110)	0.24	—	—
**External validation**				
Largest longitudinal	—	—	−0.050 (−0.131, 0.032)	0.23
18°‐interval	0.080 (0.000, 0.158)	0.048	0.029 (0.014, 0.046)	< 0.001
9°‐interval	0.084 (0.003, 0.163)	0.038	0.034 (0.017, 0.053)	< 0.001
6°‐interval	0.050 (−0.032, 0.131)	0.23	—	—

Abbreviations: AUC = area under the receiver operating characteristic curve.

To assess whether the model's predictive performance was consistent across tumor differentiation grades, the 9°‐interval model was further evaluated in the training and internal validation cohorts stratified by differentiation status (well/moderate vs. poor/moderate‐poor) (Table S2). The external validation cohort was excluded as all patients with poor/moderate‐poor differentiation (*n* = 7) were non‐pCR cases. No significant differences in predictive performance were observed between subgroups (training, *p* = 0.228; internal validation, *p* = 0.303), confirming the model's consistency across differentiation grades.

### 2.4 Integration of Clinical Factors and the Deep Learning Predictive Model

To explore whether baseline clinical factors were associated with pCR and whether they could enhance the predictive performance of the aforementioned 3D‐ResNet model, univariate and multivariate logistic regression analyses were performed in the training cohort. In the univariate analysis, the deep‐learning predictive score (*p* < 0.001) and the distance from the anal verge >5.0 cm (*p* = 0.023) showed a significant association with pCR, while pretreatment T4 stage and CEA level >5 ng/mL showed marginal significance (*p = *0.052 and *p = *0.075, respectively). These variates with *p* value < 0.10 were further adopted in the multivariate analysis, which identified both the predictive score and pretreatment T4 stage as independent predictors of pCR (Table S3), with minimal correlation (*ρ* = −0.053, *p* > 0.05) between them (Figure S2). However, the predictive score demonstrated a substantially stronger association (*β* = 20.013, 95% CI 14.923–25.097) than T4 stage (*β* = −1.421, 95% CI −2.775 to −0.666). In the internal validation, the integrated model achieved an AUC of 0.92 (95% CI 0.83–0.98), identical to that of the 3D‐ResNet model. In external validation, it showed a slight degradation (integrated model: AUC 0.84 [95% CI 0.75–0.93] vs. 3D‐ResNet model: AUC 0.86 [95% CI 0.77–0.94]). Notably, in both the internal and external validation cohorts, none of the clinical variables showed a statistically significant difference between the pCR and non‐pCR groups (Table S4), indicating that the association between clinical factors and pCR was not consistent across cohorts. In summary, these findings show that clinical factors did not improve the deep‐learning model and may even reduce its external generalizability.

### 2.5 Grad‐CAM Visualization of the Model's Discriminative Regions

**FIGURE 5:**
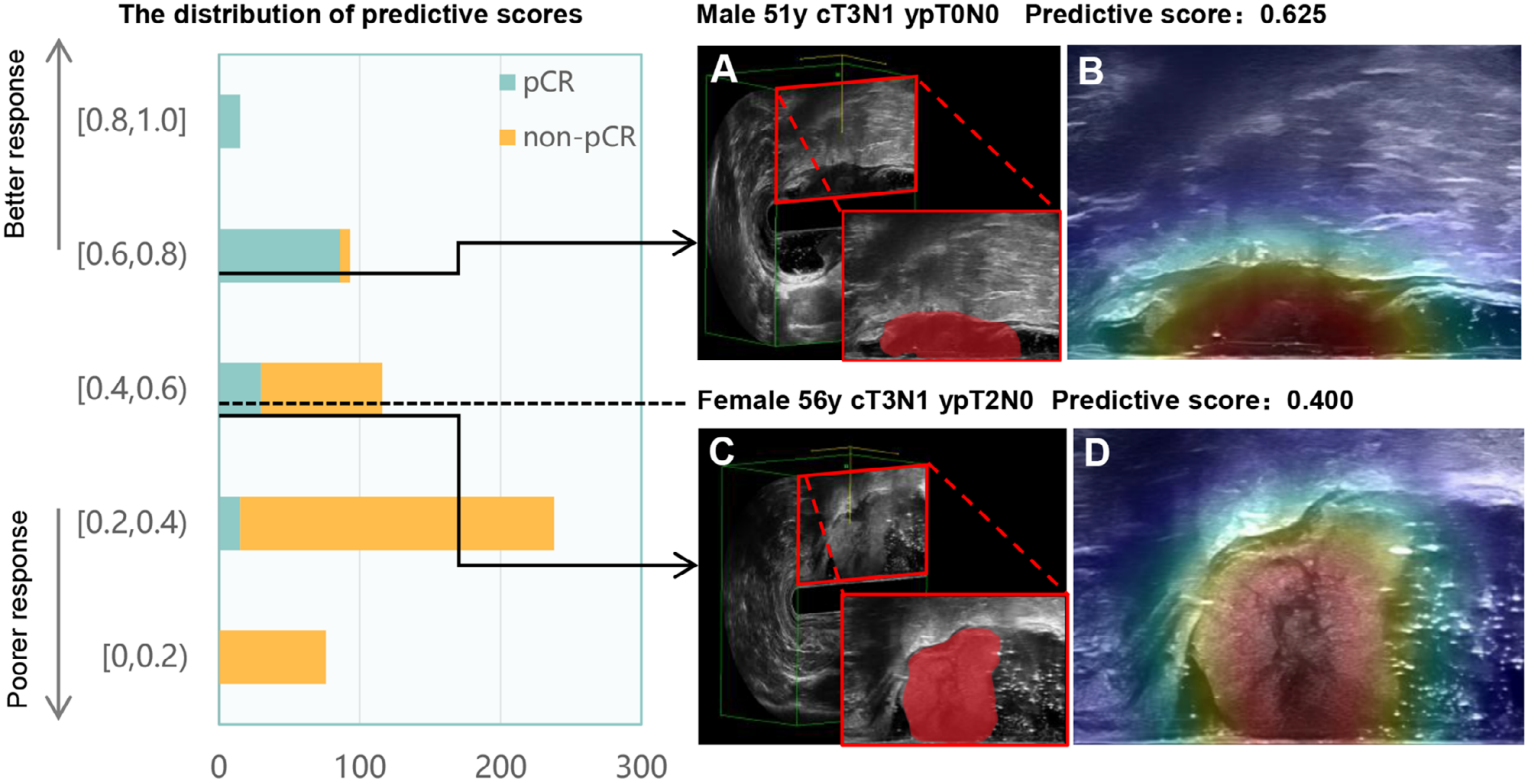
Distribution of predictive scores and representative Grad‐CAM attention maps. Left: histogram illustrating the distribution of model‐generated pCR predictive scores. The dashed horizontal line marks the optimal cut‐off value of 0.485. Right: Representative 3D TRUS frames with predicted tumor ROIs and corresponding Grad‐CAM heatmaps for a pCR case (A, B) and a non‐pCR case (C, D). In the heatmaps (B and D), the color‐coded activation patterns (ranging from blue to red) demonstrate the model's attention distribution, with the red‐highlighted areas representing the most discriminative regions that contributed significantly to the model's decision‐making process. Grad‐CAM = gradient‐weighted class activation map; pCR = pathological complete response; ROI = regions of interest; TRUS = transrectal ultrasound.

To visually interpret the decision‐making basis of the predictive model, gradient‐weighted class activation mapping (Grad‐CAM) was applied to generate heatmaps. The distribution of the model‐generated pCR possibilities across the cohorts, as well as two representative cases from pCR and non‐pCR groups, respectively, is shown in Figure 5. In a 51‐year‐old male patient with clinical T3N1 rectal cancer, the model yielded a predicted score of 0.625 (above the 0.485 cut‐off), and this patient ultimately achieved pCR following neoadjuvant therapy (Figure 5A,B). In contrast, a 56‐year‐old female patient with the same clinical T3N1 stage had a predicted score of 0.400 (below the cut‐off) and did not achieve pCR (Figure 5C, D). As observed in both cases, the most discriminative regions that contributed significantly to the predictive model showed strong spatial overlap with the rectal lesion, which were especially confined to the intra‐tumoral area, focusing on heterogeneous internal echo patterns rather than peritumoral tissues or tumor boundary. Notably, the model does not prioritize specific echo intensity (e.g., hypo‐ or hyper‐echoic); instead, it consistently identifies the tumor core itself as the key discriminative region, irrespective of its echogenicity.

To further examine whether sampling interval affects spatial attention, Grad‐CAM analyses on models trained with different intervals (6°, 9°, 18°, and single‐frame) were also performed. Heatmaps showed that all models consistently focused on heterogeneous internal echo patterns within the tumor core (Figure S3). Taken together, the model primarily relies on intra‐tumoral heterogeneity, and this attention pattern is robust across sampling intervals.

### 2.6 Association of Predictive Model With Long‐Term Prognosis

To explore whether the model‐predicted pCR status was associated with long‐term prognosis, the follow‐up data for 435 patients from Center 1 was collected. The median follow‐up duration was 30 months with an interquartile range (IQR) of 19.0–53.8 months. Kaplan–Meier analyses were performed for both disease‐free survival (DFS) and overall survival (OS) in the training and internal validation cohorts, respectively (Figures S4 and S5). Patients were stratified based on (a) actual pathological response (pCR vs. non‐pCR) and (b) response predicted by the deep learning model (predicted pCR vs. predicted non‐pCR).

In the training cohort, stratification by both actual and predicted pCR status yielded a significant difference in DFS (log‐rank *p* = 0.006 and *p* = 0.020, respectively). However, for each stratification, no significant differences were found for OS in the training cohort, nor for DFS or OS in the internal validation cohort (log‐rank *p* > 0.05). Overall, the model‐predicted pCR status showed limited association with long‐term prognosis.

## 3 Discussion

The current study developed a novel 3D CNN‐based framework that automatically segments pretreatment 3D TRUS images and leverages these data to predict pCR. This framework demonstrated excellent agreement with expert annotations and robust predictive performance across multicenter cohorts, indicating that AI‐augmented 3D TRUS is a reliable tool for personalized treatment planning in rectal cancer management.

TRUS is an accessible, cost‐effective, and real‐time imaging modality for evaluating rectal tumor, capable of visualizing internal architectural features of the tumor and assessing the depth of rectal wall invasion. However, the application of the conventional 2D TRUS is hampered by inadequate spatial contextualization and operator‐dependent variability. 3D TRUS overcomes these limitations by providing superior spatial comprehension and real‐time recording [16]. Crucially, 3D TRUS generates a stable, operator‐independent volumetric dataset that can be reviewed from any orientation, thereby ensuring repeatable observations and simplifying long‐term data preservation, paving its way for AI applications. Previous studies also demonstrated that dynamic videos or 3D data capturing tumor spatial patterns tend to yield better performance in AI‐assisted diagnostic models across multiple malignancies compared to static 2D images [20, 21, 22]. Based on these technical advantages, 3D TRUS data were selected for the development of the predictive model in the present study.

This study constructed the first deep learning model based on pretreatment 3D TRUS data to predict pCR in patients with LARC. The predictive model employing a sampling interval of 9° demonstrated excellent performance (AUCs 0.98, 0.92, and 0.85 in training, internal, and external validation, respectively). Earlier development in the field primarily focused on MRI data. Previous radiomic models using multiparametric MRI data achieved AUCs ranging from 0.77 to 0.84 in predicting pCR, superior to single‐modality MRI models [9, 10]. A subsequent multicenter study incorporated MRI radiomics signatures and clinical factors, and the model yielded AUCs up to 0.79 [11]. Another model based on cT stage, cN stage, and MRI radiomics features achieved AUCs of 0.75 and 0.83 in European and Chinese cohorts, respectively, demonstrating strong generalizability across intercontinental population [12]. More recently, deep learning methods have shown superior potential, with an MRI‐based model incorporating deep learning and sub‐regional radiomics achieving exceptional performance (AUCs exceeding 0.90 across multiple cohorts) [13]. In our study, the proposed deep learning model utilizing 3D TRUS data demonstrated comparable, if not superior, performance to existing MRI‐based AI models (as summarized in Table S5). This favorable performance may be attributed to the unique spatial and morphological information captured by 3D TRUS. These results suggest that 3D TRUS‐based deep learning models can provide competitive predictive performance while offering practical advantages in terms of accessibility and cost‐effectiveness.

A key methodological finding was that sampling intervals of 9° and 18° were found to demonstrate superior performance compared to using a single representative frame or a smaller interval of 6° (AUCs 0.92 vs. 0.79–0.83 in internal validation; AUCs 0.85–0.86 vs. 0.77–0.82 in external validation). As the different sampling intervals did not alter the model's spatial attention (Figure S3), this factor could not explain why the 9° and 18° sampling intervals showed the best performance. Nonetheless, this performance pattern suggests that strategic frame sampling may effectively balance information density against computational complexity. We hypothesize that an overly sparse interval (e.g., single‐frame) risks undersampling and may fail to capture the three‐dimensional heterogeneity of tumor architecture, while an excessively dense interval (e.g., 6°) introduces redundancy that can lead to overfitting and compromised generalizability [23, 24]. The 9° and 18° sampling intervals may strike an optimal balance by retaining sufficient cross‐sectional views to reflect intratumoral heterogeneity, while avoiding excessive redundancy. This observation aligns with prior research in prostate cancer diagnosis, where optimized sampling intervals outperformed the use of all available frames [25]. These findings provide valuable guidance for future 3D TRUS‐based model development, emphasizing that appropriate data sampling strategies are crucial for optimizing model generalizability and efficiency, although the underlying mechanisms of sampling interval selection need further exploration.

Additionally, integration of clinical factors did not enhance 3D TRUS model performance in this study. Previous studies have identified several clinical predictors of pCR, including earlier stage, smaller size, node‐negative status, low or middle tumor location, and normal baseline CEA levels [26, 27, 28, 29]. However, given the substantial difference in model contribution between the deep‐learning model‐derived predictive score (*β* = 20.013) and pretreatment T4 stage (*β* = −1.421) in the integrated model, the limited improvement in model performance can be primarily attributed to the deep learning model's inherently high baseline accuracy, which left limited potential for further enhancement from additional variables. Furthermore, in both the internal and the external validation datasets, none of the clinical variables exhibited a statistically significant difference between pCR and non‐pCR groups, indicating relatively weak and non‐reproducible univariate associations with pCR. These results are consistent with the findings of prior studies based on MRI imaging. Although some of these factors have been incorporated into MRI‐based predictive models, the inclusion of clinicopathological factors did not significantly improve model performance, and may compromise it in external validation [10, 11].

To enhance model interpretability, Grad‐CAM method was employed to visualize the regions driving predictive decisions in the model (Figure 5). The resulting heat map revealed that the focus areas were consistent with the tumor core, rather than the peripheral region. Furthermore, this focus pattern remained robust and was not significantly influenced by the sampling interval (Figure S3). These findings corroborate a prior radiomics study where central tumor feature (excluding the periphery) on T2WI/DWI images predicted treatment response in rectal cancer better than whole‐tumor features (AUC 0.84 vs. 0.69, *p = *0.008) [30]. Although a smaller MRI‐based study (*n* = 72) suggested that incorporating tumor border features could further improve the performance of the model based on tumor core features and clinical factors (AUC 0.79 vs. 0.69) [31], the current results indicate that the tumor core may contain more biologically relevant information for pCR prediction—a hypothesis worthy of further investigation.

Notably, in the segmentation component of this framework, the semi‐supervised learning (SSL)‐based segmentation model demonstrated strong agreement with manual annotations, achieving a Dice score of 0.89 on TRUS images of rectal tumors. This performance surpassed that of the standard U‐Net model (Dice score 0.86) and proved comparable to previously reported MRI‐based segmentation approaches (Dice score 0.59–0.84) [32, 33, 34, 35, 36]. The high‐quality automatic segmentation provided by this component was essential for enabling subsequent accurate pCR prediction, as it ensured consistent tumor region identification across the multicenter dataset. Previous studies in rectal tumor segmentation have predominantly utilized MRI‐based approaches. An early CNN‐based model developed for T2‐weighted and diffusion‐weighted MRI produced median Dice scores of 0.68–0.70 [32]. A subsequent U‐Net‐based model developed for T2‐weighted MRI segmentation showed comparable performance to manual annotation (Dice score 0.74 vs. 0.71) [33]. Further application of U‐Net to MRI data resulted in varying Dice scores of 0.77 and 0.59 across datasets from different institutions, underscoring how model generalization can be affected by multiple confounding factors [35]. More recently, a segmentation model integrating ResUNet and attention mechanisms achieved the current best‐reported Dice score of 0.84, outperforming a conventional U‐Net model (Dice score 0.63) [36]. These previous findings, combined with our current results, indicate that SSL methodologies offer viable pathways for enhancing segmentation accuracy. The demonstrated efficacy of CNNs in segmenting rectal tumors from ultrasound images laid a solid foundation for building large‐scale rectal tumor image databases, and thus, enabled the training of 3D TRUS‐based deep learning models.

To explore the prognostic relevance of the model's predictions, the association between predicted pCR status and long‐term outcomes were analyzed. In the training cohort, stratification by both actual and model‐predicted pCR status yielded significant differences in DFS (log‐rank *p* < 0.05), suggesting that the biological features captured by the model may be associated with early recurrence risk. However, no significant stratification was observed for DFS, which may be attributed to the smaller sample size. For OS, no significant differences were observed in either cohort. This might stem from a relatively weak correlation between pCR and 5‐year OS [37, 38], which is susceptible to confounding by post‐recurrence interventions and non‐cancer mortality. The model's prognostic utility for survival outcomes requires further validation in larger cohorts with extended follow‐up.

There are some limitations in this study. First, the model was developed and validated exclusively using 3D TRUS data acquired from a single ultrasound system across all participating centers due to the fact that only the B‐K Medical ultrasound system was equipped with a transrectal 3D volume probe during the data collection period. Although this approach ensured internal consistency in image acquisition, it may restrict the generalizability of the model to data acquired from other manufacturers. Future studies should therefore incorporate cross‐device validation using images from diverse ultrasound platforms from sufficiently large patient cohorts to evaluate and enhance the model's robustness for broader clinical application. Second, the predictive model is currently based solely on pretreatment 3D TRUS images, which limits its ability to reflect dynamic biological changes during therapy. Evidence suggests that integrating post‐treatment imaging with pretreatment data improves predictive accuracy in rectal cancer [39, 40], and that incorporating mid‐treatment images (acquired after a few cycles of therapy) enhances prediction in other malignancies [41]. Therefore, future work should aim to develop a dynamic prediction model that integrates multi‐timepoint imaging within a unified serial framework, which may capture temporal evolution and early response patterns and bring more adaptive and timely prognostic insight. Another key limitation is the inability to directly correlate Grad‐CAM activation regions with postoperative histopathology, which reduces the biological interpretability of the predictive model. This limitation arises from the temporal discrepancy between pretreatment ultrasound imaging and post‐treatment pathological specimens, as neoadjuvant therapy induces substantial tumor regression and fibrosis, fundamentally altering tissue architecture and precluding reliable spatial correspondence. Last, although the 9° and 18° sampling intervals yielded optimal model performance, the underlying mechanisms remain incompletely understood. Further investigation is warranted to elucidate how sampling interval interacts with information density and tumor heterogeneity to influence model efficacy.

In conclusion, to explore the predictive value of 3D TRUS data for the treatment response following neoadjuvant therapy in rectal cancer patients, we developed a 3D CNN framework incorporating an automatic segmentation model and a predictive model, which combines high diagnostic accuracy with minimal manual preprocessing, offering practical advantages for clinical application.

## 4 Materials and Methods

### 4.1 Ethics

This study was approved by the Institutional Review Board of Sun Yat‐Sen University Cancer Center (prospective study approval: C2019‐010‐01; retrospective study approval: B2025‐417‐01). The prospective study was registered at https://www.chictr.org.cn (No. ChiCTR1900023969). For the prospective part, written informed consent was obtained from all patients; for the retrospective part, the requirements for informed consent were waived. The study protocol was reviewed and approved by the Institutional Review Board of all participating hospitals. All patient data were de‐identified by removing direct personal identifiers and assigning unique study codes prior to analysis.

### 4.2 Patient Enrollment

As shown in Figure 1, patients with rectal cancer from the Sun Yat‐Sen University Cancer Center (Center 1), between October 2019 and June 2024, were prospectively included. To expand the sample size, eligible patients from the same hospital between April 2016 and September 2019 were retrospectively included. These patients were included as the training and the internal validation cohorts. Eligible patients from Fujian Cancer Hospital (Center 2), Yunnan Cancer Hospital (Center 3), The Sixth Affiliated Hospital of Sun Yat‐sen University (Center 4), and Chongqing University Cancer Hospital (Center 5) were prospectively included as the external validation cohort.

All participating institutions followed the same inclusion and exclusion criteria. For prospective cohorts, the inclusion criteria included (a) patients with rectal lesions pathologically confirmed as rectal carcinoma, (b) baseline clinical stage T3‐4 or any T with N+ confirmed by MRI or TRUS, and (c) potential candidates for complete neoadjuvant therapy and total mesorectal excision surgery. The exclusion criteria included (a) non‐surgery approach following neoadjuvant therapy, (b) incomplete neoadjuvant therapy, and (c) missing 3D TRUS data. For retrospective cohorts, eligible patients with rectal cancer under the same standard as the prospective cohorts were included.

### 4.3 TRUS Examination Protocol

All TRUS examinations across the five centers were performed using an Ultraview 800 ultrasound system (B‐K Medical) with a rotational 360° transducer (6–12 MHz, Type 8838). All ultrasound systems were calibrated prior to the patient enrollment, with routine calibration performed biannually in accordance with the manufacturer's operational manual. Before the examination, patients received a cleansing enema and were placed in the left lateral position. A digital rectal examination was performed to locate the lesion. Then, 50–100 mL of ultrasound coupling gel was injected into the rectum to reduce gas‐related artifacts. The transducer was positioned at the target lesion site. Through an automatic 360° rotation, the system captured a full circumferential scan of the rectal tumor and bowel wall at the same level. A 3D volume was reconstructed from over 800 transverse grayscale ultrasound images and stored in the database.

At each participating center, board‐certified radiologists with more than 5 years of clinical experience served as the operators responsible for performing TRUS and acquiring images, each having prior experience with more than 30 3D‐TRUS examinations. All the operators completed a standardized training program aimed at ensuring homogeneity in image acquisition technique, and formal patient enrollment commenced only after each operator submitted five practice cases meeting predefined quality criteria.

### 4.4 Neoadjuvant Treatment Response Assessment

The resected specimens after surgery following neoadjuvant therapies were staged according to the International Union Against Cancer TNM staging system (detailed in Table S6) [42]. The absence of any residual cancer cells was regarded as pCR.

### 4.5 Tumor Segmentation Model Development and Validation

To reduce the redundant medical image annotation task, semi‐supervised learning (SSL) method [43] was adopted. A standard U‐Net model was also trained for efficiency comparison. 3D TRUS data were decomposed into frames of grayscale TRUS images to construct a large dataset for segmentation. Based on tumor size, six to 40 frames were evenly extracted from each 3D volume. A randomly selected subset of 142 TRUS data was annotated by one radiologist (L.M.) with 12‐year experience in rectal tumor diagnosis and divided into training and validation sets at an 8:2 ratio. The training dataset was further augmented with two times its volume of unlabeled data for the segmentation model development.

A dual‐branch SSL framework was tailored for medical image segmentation, comprising a shared encoder and two task‐specific decoders. This design promotes representation sharing while enabling differentiated learning for labeled and unlabeled data.

Branch 1 receives labeled samples $x_{l}$ and learns under supervision from ground truth labels $y_{l}$. This branch also processes unlabeled images $x_{u}$ to generate pseudo‐labels ${\hat{y}}_{u}$. The loss of the branch can be formulated as:

$$\;L_{\sup}=\mathrm{DiceLoss}\left(f_{1}\left(E\left(x_{l}\right)\right),y_{l}\right)$$

Branch 2 is fed with augmented versions of the unlabeled images ${\hat{x}}_{u}$, and trained using the pseudo‐labels produced by Branch 1: $\;{\hat{y}}_{u}=f_{1}\left(E(x_{u})\right)$. The loss of the branch can be formulated as:

$$\;L_{\mathrm{unsup}}=\mathrm{DiceLoss}\left(f_{2}\left(E\left({\hat{x}}_{u}\right)\right),{\hat{y}}_{u}\right)$$

The total loss is:

$$\;L_{\mathrm{total}}=L_{\sup}+\lambda \cdot L_{\mathrm{unsup}}$$

Here, λ is a balancing coefficient, E(·) denotes the shared encoder, $f_{1}\left(\cdot \right)$ and $\;f_{2}\left(\cdot \right)$ are the two decoders, and ${\hat{x}}_{u}$ is the weakly augmented version of the unlabeled image. Through iterative experimental testing, the balancing coefficient λ was optimized, and a value of 2 yielded the optimal model performance. Figure 2B shows the semi‐supervised segmentation model.

### 4.6 PCR Predictive Model Development and Validation

The patients from SYSUCC were randomly assigned to the training and internal validation cohorts (8:2 ratio) using a computer‐generated random sequence produced by Python (NumPy library, version 1.24.3). Stratified randomization was not employed. The external validation cohort consisted of consecutive patients from the participating hospitals.

The predictive model was first pretrained using large‐scale unlabeled ultrasound datasets, including thyroid and breast tumors [44, 45, 46, 47], with a self‐supervised contrastive learning algorithm [43]. This helps to learn transferable robust features and enhances the model's generalization capability across multi‐center datasets.

To explore the optimal data volume for pCR prediction, different sampling intervals ranging from 6° to 360° were applied to each 3D TRUS data to extract different numbers of 2D frames. The sampling intervals of 6°, 9°, and 18°, respectively, correspond to 60, 40, and 20 frames evenly distributed in a 360° transrectal scan, and the interval of 360° indicated the use of a single representative frame, defined as the lesion's largest longitudinal section. To mitigate potential segmentation inaccuracies, bounding boxes were expanded by 4% in all spatial directions, ensuring coverage of tumor areas. The expanded regions of interest (ROIs) were then cropped from the original 3D volume, resampled to a uniform size, and fed into the downstream classification network. The detailed preprocessing process of each frame is summarized in Appendix S1.

A standard 3D‐ResNet architecture was selected as the backbone for the classification network. A convolutional block attention module (CBAM) was inserted before the global average pooling layer to emphasize prognostically relevant regions. CBAM sequentially applies two sub‐modules, channel and spatial attention. Channel attention reweights features via aggregated pooling descriptors, while spatial attention generates a focus map through convolution over channel‐refined features. Together, the two sub‐modules enhance sensitivity to lesion characteristics. The refined features are then pooled and passed through a fully connected layer for final prediction. The classification network is trained using the standard cross‐entropy loss, defined as:

$$\;L_{cls}=-\sum_{i=1}^{C}y_{c}^{\left(i\right)}\log\left({\hat{y}}_{c}^{\left(i\right)}\right)$$

where C denotes the number of categories, ${\hat{y}}_{c}^{\left(i\right)}$ is the predicted probability for class i, and $y_{c}^{\left(i\right)}$ is the one‐hot encoded ground truth label. The model was trained with a batch size of 16. The learning rate was initialized at 0.002, and training was conducted over 2000 iterations. A dropout regularization coefficient of 0.5 was applied throughout. Figure 2C shows the predictive model for pCR in rectal cancer. All experiments in this study were conducted using the PyTorch 1.13.1 (https://pytorch.org/get‐started/previous‐versions/) framework with CUDA 11.7 (https://developer.nvidia.com/cuda‐11‐7‐0‐download‐archive).

### 4.7 Heat Map Generation

To improve the interpretability of the predictive model, gradient‐weighted class activation mapping (Grad‐CAM) was applied to each individual input image frame to generate corresponding heat maps [48]. These visualizations highlight the most informative regions contributing to predicting the treatment response, reflecting the pixel‐level importance across the input space. The images were generated using OpenCV 4.7.0 (https://github.com/opencv/opencv/releases/tag/4.7.0) and Matplotlib 3.6.2 (https://matplotlib.org/3.6.2/users/installing.html).

### 4.8 Statistical Analysis

The differences in the clinicopathological characteristics between patients in different cohorts were compared using the independent *t*‐test or Mann–Whitney *U* test for continuous variables, and chi‐squared test for categorical variables. For the segmentation model, the IoU and Dice score were calculated to assess the accuracy of segmentation. For each predictive model, the performance was evaluated and compared using receiver operating characteristic (ROC) curves. The optimal cut‐off values were calculated by maximizing the Youden index in the training dataset. The same cut‐off values were applied to the internal and external validation datasets. The accuracy, sensitivity, specificity, positive predictive value (PPV), and negative predictive value (NPV) were calculated using a generalized estimating equation. Their 95% confidence intervals (95% CIs) were calculated using the Wilson score interval method. The differences of areas under curves (AUCs) among cohorts were assessed using bootstrap resampling method. A total of 1000 bootstrap samples were generated by randomly sampling with replacement. For each bootstrap sample, the AUCs of each cohort and the △AUCs between groups were calculated. The resulting distributions were used to derive 95% CIs and estimate two‐tailed *p* values. A two‐tailed *p* < 0.05 was defined as significant. The multivariable logistic regression was used to develop the integrated model combining the deep learning model and clinical factors. Statistical analyses were performed using SPSS (version 26.0, https://www.ibm.com/products/spss) and R software (version 4.3.1, http://www.R‐project.org).

## Author Contributions

Conceptualization: Min Liu, Ruohan Guo, and Jianhua Zhou. Data Curation: Min Liu, Yiwen Yu, and Ruohan Guo. Formal analysis: Yiwen Yu and Ying Liao. Funding acquisition: Min Liu and Jianhua Zhou. Investigation: Yiwen Yu, Xuebin Zou, Ying Liao, and Juan Fu. Methodology: Min Liu and Ruohan Guo. Project administration: Anhua Li and Jianhua Zhou. Resources: Anhua Li, Jianhua Zhou, Lina Tang, Xiaomao Luo, Guangjian Liu, and Fang Li. Software: Lichao Mou, Xing Zhao, and Jingliang Hu. Supervision: Anhua Li, Jianwei Wang, and Jianhua Zhou. Validation: Min Liu, Xuebin Zou, and Yiwen Yu. Visualization: Yiwen Yu, Lichao Mou, and Xing Zhao. Writing – original draft: Min Liu, Yiwen Yu, Xuebin Zou, and Ying Liao. Writing – review and editing: Anhua Li, Jianwei Wang, Ruohan Guo, and Jianhua Zhou. All authors have read and approved the final manuscript.

## Funding

This study was supported by the National Natural Science Foundation of China (Grant numbers: 82320108011 and 82302209) and the Natural Science Foundation of Guangdong Province (Grant number: 2024A1515012000). The funding source had no involvement in study design, data collection, data analysis, or manuscript preparation or approval.

## Ethics Statement

This study was approved by the Institutional Review Board of Sun Yat‐Sen University Cancer Center (approval numbers: C2019‐010‐01 for the prospective component and B2025‐417‐01 for the retrospective component) and was conducted in accordance with the Declaration of Helsinki. The prospective study was registered at the Chinese Clinical Trial Registry (https://www.chictr.org.cn) under registration number ChiCTR1900023969. Written informed consent was obtained from all prospectively enrolled patients; the requirements for informed consent were waived for retrospectively enrolled patients.

## Conflicts of Interest

Lichao Mou, Xing Zhao, and Jingliang Hu are employees of MedAI Technology (Wuxi) Co., Ltd., but have no potential relevant financial or non‐financial interests to disclose. The other authors have no conflicts of interest to declare.

## Supporting information




**Supporting File 1**: mco270982‐sup‐0001‐SuppMat.docx.

**Supporting File 2**: mco270982‐sup‐0002‐CodeS1.py.

**Supporting File 3**: mco270982‐sup‐0003‐CodeS2.py.

## Data Availability

The data that support the findings of this study are not publicly available due to privacy restrictions. The data may be obtained from the author, Min Liu (liumin@sysucc.org.cn), upon reasonable request approved by the institutional review board. See Appendix S2 for details. The code for the semi‐supervised segmentation model and the predictive 3D‐ResNet model that support the findings of this study are available in the Supporting Information of this article (Code S1 and Code S2).

## References

[1] F. Bray , M. Laversanne , H. Sung , et al., “Global Cancer Statistics 2022: GLOBOCAN Estimates of Incidence and Mortality Worldwide for 36 Cancers in 185 Countries,” CA: A Cancer Journal for Clinicians 74, no. 3 (2024): 229–263. 38572751 10.3322/caac.21834

[2] R. L. Siegel , N. S. Wagle , A. Cercek , R. A. Smith , and A. Jemal , “Colorectal Cancer Statistics,” CA: A Cancer Journal for Clinicians 73, no. 3 (2023): 233–254. 36856579 10.3322/caac.21772

[3] M. Maas , P. J. Nelemans , V. Valentini , et al., “Long‐Term Outcome in Patients With a Pathological Complete Response After Chemoradiation for Rectal Cancer: A Pooled Analysis of Individual Patient Data,”Lancet Oncology 11, no. 9 (2010): 835–844. 20692872 10.1016/S1470-2045(10)70172-8

[4] A. Kasi , S. Abbasi , S. Handa , et al., “Total Neoadjuvant Therapy vs Standard Therapy in Locally Advanced Rectal Cancer,” JAMA Network Open 3, no. 12 (2020): e2030097. 33326026 10.1001/jamanetworkopen.2020.30097 PMC7745099

[5] M. J. M. van der Valk , D. E. Hilling , E. Bastiaannet , G. L. Beets , N. L. Figueiredo , et al., “Long‐Term Outcomes of Clinical Complete Responders After Neoadjuvant Treatment for Rectal Cancer in the International Watch & Wait Database (IWWD): An International Multicentre Registry Study,” Lancet 391, no. 10139 (2018): 2537–2545. 29976470 10.1016/S0140-6736(18)31078-X

[6] J. J. Smith , P. Strombom , O. S. Chow , et al., “Assessment of a Watch‐and‐Wait Strategy for Rectal Cancer in Patients With a Complete Response After Neoadjuvant Therapy,” JAMA Oncology 5, no. 4 (2019): e185896. 30629084 10.1001/jamaoncol.2018.5896 PMC6459120

[7] C. Cerdan‐Santacruz , G. P. São Julião , B. B. Vailati , L. Corbi , A. Habr‐Gama , and R. O. Perez , “Watch and Wait Approach for Rectal Cancer,” Journal of Clinical Medicine 12, no. 8 (2023): 2873. 37109210 10.3390/jcm12082873 PMC10143332

[8] R. J. Gillies , P. E. Kinahan , and H. H. Radiomics , “Radiomics: Images Are More Than Pictures, They Are Data,” Radiology 278, no. 2 (2016): 563–577. 26579733 10.1148/radiol.2015151169 PMC4734157

[9] K. Nie , L. Shi , Q. Chen , X. Hu , S. K. Jabbour , and N. Yue , "Rectal Cancer: Assessment of Neoadjuvant Chemoradiation Outcome Based on Radiomics of Multiparametric MRI," Clinical Cancer Research: An Official Journal of the *American Association for Cancer Research* (2016): 5256–5264. 10.1158/1078-0432.CCR-15-2997 PMC10916000 27185368

[10] X. Zhou , Y. Yi , Z. Liu , et al., “Radiomics‐Based Pretherapeutic Prediction of Non‐Response to Neoadjuvant Therapy in Locally Advanced Rectal Cancer,” Annals of Surgical Oncology 26, no. 6 (2019): 1676–1684. 30887373 10.1245/s10434-019-07300-3 PMC6510882

[11] M. Song , S. Li , H. Wang , et al., “MRI Radiomics Independent of Clinical Baseline Characteristics and Neoadjuvant Treatment Modalities Predicts Response to Neoadjuvant Therapy in Rectal Cancer,” British Journal of Cancer 127, no. 2 (2022): 249–257. 35368044 10.1038/s41416-022-01786-7 PMC9296479

[12] L. Boldrini , J. Lenkowicz , L. C. Orlandini , et al., “Applicability of a Pathological Complete Response Magnetic Resonance‐Based Radiomics Model for Locally Advanced Rectal Cancer in Intercontinental Cohort,” Radiation Oncology 17, no. 1 (2022): 78. 35428267 10.1186/s13014-022-02048-9 PMC9013126

[13] X. Wu , J. Wang , C. Chen , et al., “Integration of Deep Learning and Sub‐Regional Radiomics Improves the Prediction of Pathological Complete Response to Neoadjuvant Chemoradiotherapy in Locally Advanced Rectal Cancer Patients,” Academic Radiology 32, no. 6 (2025): 3384–3396. 39809603 10.1016/j.acra.2024.12.049

[14] A. D. R. Grey , M. J. Connor , J. Tam , and T. Loch , “Can Transrectal Prostate Ultrasound Compete With Multiparametric MRI in the Detection of Clinically Significant Prostate Cancer?,” Translational Andrology and Urology 9, no. 3 (2020): 1492–1500. 32676436 10.21037/tau.2020.02.26 PMC7354342

[15] A. M. Lahoti , A. P. Dhok , C. R. Rantnaparkhi , J. S. Rawat , N. U. Chandak , and H. S. Tawari , “Role of Magnetic Resonance Imaging, Magnetic Resonance Spectroscopy and Transrectal Ultrasound in Evaluation of Prostatic Pathologies With Focus on Prostate Cancer,” Polish Journal of Radiology 82 (2017): 827–836. 29657651 10.12659/PJR.903958 PMC5894040

[16] R. A. Pinto , I. J. Corrêa Neto , S. C. Nahas , C. S. Rizkalah Nahas , C. F. Sparapan Marques , U. Ribeiro Junior , et al., “Efficacy of 3‐Dimensional Endorectal Ultrasound for Staging Early Extraperitoneal Rectal Neoplasms,” Diseases of the Colon & Rectum 60, no. 5 (2017): 488–496. 28383448 10.1097/DCR.0000000000000781

[17] A. E. Ilesanmi , T. O. Ilesanmi , and B. O. Ajayi , “Reviewing 3D Convolutional Neural Network Approaches for Medical Image Segmentation,” Heliyon 10, no. 6 (2024): e27398. 38496891 10.1016/j.heliyon.2024.e27398 PMC10944240

[18] Y. Zhou , C. Chen , J. Yao , et al., “A Deep Learning Based Ultrasound Diagnostic Tool Driven by 3D Visualization of Thyroid Nodules,” NPJ Digital Medicine 8, no. 1 (2025): 126. 40016505 10.1038/s41746-025-01455-y PMC11868480

[19] J. Zhang , J. Wu , X. S. Zhou , F. Shi , and D. Shen , “Recent Advancements in Artificial Intelligence for Breast Cancer: Image Augmentation, Segmentation, Diagnosis, and Prognosis Approaches,” Seminars in Cancer Biology 96 (2023): 11–25. 37704183 10.1016/j.semcancer.2023.09.001

[20] X. Liu , T. Wang , G. Zhang , et al., “Two‐Dimensional and Three‐Dimensional T2 Weighted Imaging‐Based Radiomic Signatures for the Preoperative Discrimination of Ovarian Borderline Tumors and Malignant Tumors,” Journal of Ovarian Research 15, no. 1 (2022): 22. 35115022 10.1186/s13048-022-00943-z PMC8815217

[21] G. Zhao , D. Kong , X. Xu , S. Hu , Z. Li , and J. Tian , “Deep Learning‐Based Classification of Breast Lesions Using Dynamic Ultrasound Video,” European Journal of Radiology 165 (2023): 110885. 37290361 10.1016/j.ejrad.2023.110885

[22] T. Qian , Y. Zhou , J. Yao , et al., “Deep Learning Based Analysis of Dynamic Video Ultrasonography for Predicting Cervical Lymph Node Metastasis in Papillary Thyroid Carcinoma,” Endocrine 87, no. 3 (2025): 1060–1069. 39556263 10.1007/s12020-024-04091-w

[23] V. S. Parekh and M. A. Jacobs , “Multiparametric Radiomics Methods for Breast Cancer Tissue Characterization Using Radiological Imaging,” Breast Cancer Research and Treatment 180, no. 2 (2020): 407–421. 32020435 10.1007/s10549-020-05533-5 PMC7066290

[24] V. Berisha , C. Krantsevich , P. R. Hahn , et al., “Digital Medicine and the Curse of Dimensionality,” NPJ Digital Medicine 4, no. 1 (2021): 153. 34711924 10.1038/s41746-021-00521-5 PMC8553745

[25] Y.‐K. Sun , B.‐Y. Zhou , Y. Miao , et al., “Three‐Dimensional Convolutional Neural Network Model to Identify Clinically Significant Prostate Cancer in Transrectal Ultrasound Videos: A Prospective, Multi‐institutional, Diagnostic Study,” EClinicalMedicine 60 (2023): 102027. 37333662 10.1016/j.eclinm.2023.102027 PMC10276260

[26] Y. Huang , D. Lee , and C. Young , “Predictors for Complete Pathological Response for Stage II and III Rectal Cancer Following Neoadjuvant Therapy—A Systematic Review and Meta‐Analysis,”American Journal of Surgery 220, no. 2 (2020): 300–308. 31937416 10.1016/j.amjsurg.2020.01.001

[27] C. Timmerman , L. R. Taveras , and S. Huerta , “Clinical and Molecular Diagnosis of Pathologic Complete Response in Rectal Cancer: An Update,” Expert Review of Molecular Diagnostics 18, no. 10 (2018): 887–896. 30124091 10.1080/14737159.2018.1514258

[28] S. V. Patel , C. S. Roxburgh , E. Vakiani , et al., “Distance to the Anal Verge Is Associated With Pathologic Complete Response to Neoadjuvant Therapy in Locally Advanced Rectal Cancer,” Journal of Surgical Oncology 114, no. 5 (2016): 637–641. 27641934 10.1002/jso.24358 PMC5516624

[29] Q. Zhang , J. Liang , J. Chen , S. Mei , and Z. Wang , “Predictive Factors for Pathologic Complete Response Following Neoadjuvant Chemoradiotherapy for Rectal Cancer,” Asian Pacific Journal of Cancer Prevention 22, no. 5 (2021): 1607–1611. 34048192 10.31557/APJCP.2021.22.5.1607 PMC8408379

[30] N. Korsavidou Hult , S. Tarai , K. Hammarström , et al., “Inclusion of Tumor Periphery in Radiomics Analysis of Magnetic Resonance Images Does Not Improve Predictions of Preoperative Therapy Response in Patients With Rectal Cancer,” Abdominal Radiology 51, no. 3 (2026): 1116–1128. 39907722 10.1007/s00261-025-04815-0 PMC12971816

[31] A. Delli Pizzi , A. M. Chiarelli , P. Chiacchiaretta , et al., “MRI‐Based Clinical‐Radiomics Model Predicts Tumor Response Before Treatment in Locally Advanced Rectal Cancer,” Scientific Reports 11, no. 1 (2021): 5379. 33686147 10.1038/s41598-021-84816-3 PMC7940398

[32] S. Trebeschi , J. J. M. van Griethuysen , D. M. J. Lambregts , et al., “Deep Learning for Fully‐Automated Localization and Segmentation of Rectal Cancer on Multiparametric MR,” Scientific Reports 7, no. 1 (2017): 5301. 28706185 10.1038/s41598-017-05728-9 PMC5509680

[33] J. Wang , J. Lu , G. Qin , et al., “Technical Note: A Deep Learning‐Based Autosegmentation of Rectal Tumors in MR Images,” Medical Physics 45, no. 6 (2018): 2560–2564. 29663417 10.1002/mp.12918

[34] H. T. Zhu , X. Y. Zhang , Y. J. Shi , X. T. Li , and Y. S. Sun , “Automatic Segmentation of Rectal Tumor on Diffusion‐Weighted Images by Deep Learning With U‐Net,” Journal of Applied Clinical Medical Physics 22, no. 9 (2021): 324–331. 34343402 10.1002/acm2.13381 PMC8425941

[35] F. Knuth , I. A. Adde , B. N. Huynh , et al., “MRI‐based Automatic Segmentation of Rectal Cancer Using 2D U‐Net on Two Independent Cohorts,” Acta Oncologica 61, no. 2 (2022): 255–263. 34918621 10.1080/0284186X.2021.2013530

[36] Z. Zhang , J. Han , W. Ji , et al., “Improved Deep Learning for Automatic Localisation and Segmentation of Rectal Cancer on T2‐Weighted MRI,” Journal of Medical Radiation Sciences 71, no. 4 (2024): 509–518. 38654675 10.1002/jmrs.794 PMC11638361

[37] F. Petrelli , K. Borgonovo , M. Cabiddu , M. Ghilardi , V. Lonati , and S. Barni , “Pathologic Complete Response and Disease‐Free Survival Are Not Surrogate Endpoints for 5‐Year Survival in Rectal Cancer: An Analysis of 22 Randomized Trials,” Journal of Gastrointestinal Oncology 8, no. 1 (2017): 39–48. 28280607 10.21037/jgo.2016.11.03 PMC5334047

[38] M. Buyse , E. D. Saad , T. Burzykowski , M. M. Regan , and C. S. Sweeney , “Surrogacy Beyond Prognosis: The Importance of “Trial‐Level” Surrogacy,” Oncologist 27, no. 4 (2022): 266–271. 35380717 10.1093/oncolo/oyac006 PMC8982389

[39] C. Jin , H. Yu , J. Ke , et al., “Predicting Treatment Response From Longitudinal Images Using Multi‐Task Deep Learning,” Nature Communications 12, no. 1 (2021): 1851. 10.1038/s41467-021-22188-y PMC7994301 33767170

[40] X.‐Y. Zhang , L. Wang , H.‐T. Zhu , et al., “Predicting Rectal Cancer Response to Neoadjuvant Chemoradiotherapy Using Deep Learning of Diffusion Kurtosis MRI,” Radiology 296, no. 1 (2020): 56–64. 32315264 10.1148/radiol.2020190936

[41] W. Tang , C. Jin , Q. Kong , et al., “Development and Validation of an MRI Spatiotemporal Interaction Model for Early Noninvasive Prediction of Neoadjuvant Chemotherapy Response in Breast Cancer: A Multicentre Study,” EClinicalMedicine 85 (2025): 103298. 40584836 10.1016/j.eclinm.2025.103298 PMC12205660

[42] J. D. Brierley , M. Giuliani , B. O'Sullivan , B. Rous , and L. Van Eycken (Eds.), TNM Classification of Malignant Tumours, 9th ed. (Wiley‐Blackwell, 2025).

[43] K. He , H. Fan , Y. Wu , S. Xie , and R. Girshick , “Momentum Contrast for Unsupervised Visual Representation Learning,” in Proceedings of the IEEE/CVF Conference on Computer Vision and Pattern Recognition (IEEE, 2020), 9729–9738.

[44] H. Gong , J. Chen , G. Chen , H. Li , G. Li , and F. Chen , “Thyroid Region Prior Guided Attention for Ultrasound Segmentation of Thyroid Nodules,” Computers in Biology and Medicine 155 (2023): 106389. 36812810 10.1016/j.compbiomed.2022.106389

[45] W. Al‐Dhabyani , M. Gomaa , H. Khaled , and A. Fahmy , “Dataset of Breast Ultrasound Images,” Data in Brief 28 (2020): 104863. 31867417 10.1016/j.dib.2019.104863 PMC6906728

[46] Y. Mo , C. Han , Y. Liu , et al., “HoVer‐Trans: Anatomy‐Aware HoVer‐Transformer for ROI‐Free Breast Cancer Diagnosis in Ultrasound Images,” IEEE Transactions on Medical Imaging 42, no. 6 (2023): 1696–1706. 37018705 10.1109/TMI.2023.3236011

[47] L. Pedraza , C. Vargas , F. Narváez , O. Durán , E. Muñoz , and E. Romero , “An Open Access Thyroid Ultrasound Image Database,” in Proceedings of the 10th International Symposium on Medical Information Processing and Analysis, Vol. 9287 (SPIE, 2015), 188–193.

[48] R. R. Selvaraju , M. Cogswell , A. Das , R. Vedantam , D. Parikh , and D. Batra , “Grad‐cam: Visual Explanations from Deep Networks via Gradient‐Based Localization,” in Proceedings of the IEEE International Conference on Computer Vision (IEEE, 2017), 618–626.

