# Supplementary material for: A Deep Learning Framework Predicts Rectal Cancer Treatment Response via Pretreatment 3D Transrectal Ultrasound: A Multicenter Study
Source: MedComm (2020). 2026 Sep 9;7(9):e70982. doi: 10.1002/mco2.70982 (PMC13555799; doi:10.1002/mco2.70982)
Supplement: Supplementary file 1 [file MCO2-7-e70982-s001.docx]

**Supplementary Materials for**

A Deep Learning Framework Predicts Rectal Cancer Treatment Response

via Pre-treatment 3D Transrectal Ultrasound: A Multicenter Study

Min Liu^1#^, Yiwen Yu^1#^, Xuebin Zou^1#^, Ying Liao^1#^, Lichao Mou^2^, Xing Zhao^2^, Juan Fu^1^, Lina Tang^3^, Xiaomao Luo^4^, Guangjian Liu^5^, Fang Li^6^, Jingliang Hu^2^, Anhua Li^1*^, Jianwei Wang^1*^, Ruohan Guo^1*^, Jianhua Zhou^1*^

^1^ Department of Ultrasound, Sun Yat-Sen University Cancer Center, State Key Laboratory of Oncology in South China, Guangdong Provincial Clinical Research Center for Cancer, Guangzhou, China.

^2^ MedAI Technology (Wuxi) Co., Ltd., Wuxi, China

^3^ Department of Diagnostic Ultrasound, Fujian Cancer Hospital and Fujian Medical University Cancer Hospital, Fuzhou, China.

^4^ Department of Medical Ultrasound, Yunnan Cancer Hospital & The Third Affiliated Hospital of Kunming Medical University, Kunming, China.

^5^ Department of Ultrasound, The Sixth Affiliated Hospital, Sun Yat-Sen University, Guangzhou, China.

^6^ Department of Ultrasound, Chongqing University Cancer Hospital, School of Medicine, Chongqing University, Chongqing, China.

\# Co-first authors.

* Corresponding authors.

Corresponding authors:

Jianhua Zhou (zhoujh@sysucc.org.cn)

Ruohan Guo (guorh@sysucc.org.cn)

Jianwei Wang (wangjw@sysucc.org.cn)

Anhua Li (liah@sysucc.org.cn)

**Figure S1. The distribution of AUC values and pairwise AUC differences**

The figure displays the distribution of AUC values for predictive models across varying sampling intervals (A), along with pairwise AUC differences between models (B) in the training, the internal validation, and the external validation cohorts.

AUC = areas under the receiver operating characteristic curve; ROC = Receiver operating characteristic curve.

**Figure S2. Spearman correlation heatmap of predictive scores and clinical variables.**

This heatmap depicts the pairwise Spearman's rank correlation coefficients (ρ) among predictive scores and clinical variables that showed a *P*-value <0.10 in the univariate analysis (see Table S2), including pre-treatment T stage, distance from the anal verge, and CEA Level. Correlation strength is color-coded from white (strong negative correlation, ρ = -1) through light blue (no correlation, ρ = 0) to dark red (strong positive correlation, ρ = +1). The pairwise *P*-values were all > 0.05.

AV = anal verge; CEA = carcinoembryonic antigen.

**Figure S3. Comparison of spatial attention across pCR predictive models with different sampling intervals.**

Grad-CAM heatmaps for two representative rectal lesions with different outcomes (upper row: pCR; lower row: non-pCR) illustrate the focus of predictive models, showing that predictive models trained with sampling intervals of single-frame (A, E), 18° (B, F), 9°(C, G), and 6° (D, H) inputs all focused on similar regions—specifically, the heterogeneous internal echo patterns within the tumor core.

Grad-CAM = Gradient-weighted Class Activation Mapping; pCR = pathological complete response.

**Figure S4. Kaplan-Meier curves of disease-free survival (DFS) in the training and the internal validation cohorts.**

Patients are stratified in two distinct ways: (A, C) by the actual pathological response (pCR vs. non-pCR) and (B, D) by the deep learning model-predicted pathological response (predicted pCR vs. predicted non-pCR). Differences between survival curves were compared using the log-rank test. pCR = pathological complete response.

**Figure S5. Kaplan-Meier curves of overall survival (OS) in the training and the internal validation cohorts.**

Patients are stratified in two distinct ways: (A, C) by the actual pathological response (pCR vs. non-pCR) and (B, D) by the deep learning model-predicted pathological response (predicted pCR vs. predicted non-pCR). Differences between survival curves were compared using the log-rank test.  pCR = pathological complete response.

**Table S1.  Segmentation performance comparison between semi-supervised**

**and standard U-Net models across subgroups.**

| **Characteristic** |  | **Dice score** | ***P* value** | **IoU** | ***P* value** |
| --- | --- | --- | --- | --- | --- |
| **Pre-treatment T stage** |  |  |  |  |  |
| T1-2 (n = 42) | Semi-supervised | 0.86 ± 0.13 | <0.001 | 0.76 ± 0.15 | <0.001 |
|  | Standard | 0.83 ± 0.12 |  | 0.73 ± 0.15 |  |
| T3 (n = 370) | Semi-supervised | 0.90 ± 0.08 | <0.001 | 0.82 ± 0.10 | <0.001 |
|  | Standard | 0.86 ± 0.14 |  | 0.77 ± 0.16 |  |
| T4 (n = 178) | Semi-supervised | 0.90 ± 0.11 | <0.001 | 0.82 ± 0.14 | <0.001 |
|  | Standard | 0.87 ± 0.13 |  | 0.79 ± 0.16 |  |
| **Tumor size** |  |  |  |  |  |
| < 4.0 cm (n = 242) | Semi-supervised | 0.89 ± 0.09 | <0.001 | 0.81 ± 0.12 | <0.001 |
|  | Standard | 0.85 ± 0.16 |  | 0.76 ± 0.17 |  |
| ≥ 4.0 cm (n = 348) | Semi-supervised | 0.90 ± 0.10 | <0.001 | 0.82 ± 0.12 | <0.001 |
|  | Standard | 0.87 ± 0.11 |  | 0.78 ± 0.15 |  |
| **Distance from anal verge** |  |  |  |  |  |
| < 5.0 cm (n = 250) | Semi-supervised | 0.90 ± 0.06 | <0.001 | 0.83 ± 0.09 | <0.001 |
|  | Standard | 0.87 ± 0.08 |  | 0.78 ± 0.12 |  |
| ≥ 5.0 cm (n = 340) | Semi-supervised | 0.89 ± 0.11 | <0.001 | 0.81 ± 0.14 | <0.001 |
|  | Standard | 0.85 ± 0.16 |  | 0.77 ± 0.18 |  |

Data are presented as mean ± standard deviation. *P* values were derived from paired t-tests comparing the segmentation performance between the semi-supervised model and the standard U-Net model for each subgroup. IoU = Intersection of Union.

**Table S2. Performance of predictive models across tumors of different differentiation grades.**

|  | **AUC (95% CI)** | **Accuracy*** | **Sensitivity*** | **Specificity*** | **PPV*** | **NPV*** |
| --- | --- | --- | --- | --- | --- | --- |
| **Training cohort** |  |  |  |  |  |  |
| M/D or W/D (n=271) | 0.97 (0.95 - 1.00) | 94.5% (256/271)  [91.1% - 96.7%] | 90.3% (56/62)  [80.5% - 95.5%] | 95.6% (200/209)  [92.0% - 97.8%] | 86.2% (56/65)  [75.9% - 92.5%] | 97.1% (200/206)  [93.8% - 96.7%] |
| P/D or P-M/D (n=62) | 0.99 (0.87 - 1.00) | 93.5% (58/62)  [84.6% - 99.6%] | 94.7% (18/19)  [75.4% - 99.1%] | 93.0% (40/43)  [81.4% - 97.6%] | 85.7% (18/21)  [65.4% - 95.0%] | 97.6% (40/41)  [87.4% - 99.6%] |
| **Internal validation cohort** |  |  |  |  |  |  |
| M/D or W/D (n=72) | 0.91 (0.81 - 1.00) | 86.1% (62/72)  [76.3% - 92.3%] | 82.4% (14/17)  [59.0% - 93.8%] | 87.3% (48/55)  [76.0% - 93.7%] | 66.7% (14/21)  [45.4% - 82.8%] | 94.1% (48/51)  [84.1% - 98.0%] |
| P/D or P-M/D (n=9) | 0.86 (0.57 - 1.00) | 77.8% (7/9)  [46.7% - 99.5%] | 50.0% (1/2)  [9.5% - 90.6%] | 85.8% (6/7)  [46.7% - 99.5%] | 50.0% (1/2)  [9.5% - 90.6%] | 85.8% (6/7)  [46.7% - 99.5%] |

The external validation cohort was not included, as all poorly to moderately differentiated and poorly differentiated tumors in the external validation dataset (n=7) were non-pCR cases.

* Values are presented as point estimate % (numerator/denominator) [95% confidence interval].

AUC = areas under the receiver operating characteristic curve; CI = confidence interval; M/D = moderately differentiated; NPV = negative predictive value; P/D = poorly differentiated; P-M/D: poorly to moderately differentiated; PPV= positive predictive value; W/D: well differentiated.

**Table S3. Univariate and multivariate analysis of clinical variables and predictive risk scores in the training cohort.**

| **Variables** | **Odds ratio (95% CI)** | ***P* value** | ***β** (95% CI)** | **Odds ratio (95% CI)** | ***P* Value** |
| --- | --- | --- | --- | --- | --- |
| Age (y)* | 0.987 (0.964, 1.011) | 0.267 | - | - | - |
| Male | 0.684 (0.425, 1.102) | 0.118 | - | - | - |
| CEA > 5 ng/ml | 0.643 (0.395, 1.047) | 0.075 | - | - | - |
| CA199 > 35 U/ml | 1.586 (0.844, 2.980) | 0.149 | - | - | - |
| Tumor size (cm)* | 0.996 (0.979, 1.014) | 0.650 | - | - | - |
| Distance from anal verge > 5.0 cm | 1.821 (1.081, 3.067) | **0.023** | - | - | - |
| Pre-treatment T4 | 0.565 (0.316, 1.010) | 0.052 | -1.421(-2.775, -0.666) | 0.241 (0.062, 0.935) | 0.040 |
| Pre-treatment N+ | 0.593 (0.250, 1.404) | 0.230 | - | - | - |
| Middle or well differentiated** | 0.672 (0.365, 1.236) | 0.198 | - | - | - |
| Predictive risk score*** | 2.34×10^8^ (1.92×10^6^, 2.86×10^10^) | **<0.001** | 20.013 (14.923, 25.097) | 4.91×10^8^ (3.03×10^6^, 7.98×10^10^) | <0.001 |

* β is the regression coefficient. Variables with *p* < 0.10 in the univariate analysis were further included in the multivariate analysis.

** Fifteen lesions without applicable tumor differentiation results were not taken into account.

*** The risk score generated by the 9°-interval predictive model was adopted here.

CA199 = carbohydrate antigen 19-9; CEA = carcinoembryonic antigen; CI = Confidence interval.

**Table S4. Distribution of baseline clinical characteristics in pCR and non-pCR patients.**

|  | **Training (n = 348)** | | | **Internal Validation (n = 87)** | | | **External Validation (n = 103)** | | |
| --- | --- | --- | --- | --- | --- | --- | --- | --- | --- |
| **Characteristic** | **pCR**  **(n = 94)** | **Non-pCR**  **(n = 254)** | ***P* value** | **pCR**  **(n = 24)** | **Non-pCR**  **(n = 63)** | ***P* value** | **pCR**  **(n = 28)** | **Non-pCR**  **(n = 75)** | ***P* value** |
| **Age** (y)* | 55.7 ± 10.8 | 57.1 ± 10.2 | 0.267 | 57.9 ± 8.4 | 56.9 ± 11.1 | 0.655 | 56.2 ± 12.1 | 55.9 ± 11.6 | 0.915 |
| **Sex** |  |  | 0.118 |  |  |  |  |  | 0.793 |
| Female | 45 (47.9%) | 102 (39.6%) |  | 6 (25.0%) | 25 (39.7%) | 0.201 | 12(42.9%) | 30 (40.0%) |  |
| Male | 49 (52.1%) | 152 (61.4%) |  | 18 (75.0%) | 38 (60.3%) |  | 16 (57.1%) | 45 (60.0%) |  |
| **CEA** (ng/ml)** |  |  | 0.075 |  |  | 0.194 |  |  | 0.769 |
| > 5 | 34 (36.2%) | 119 (46.9%) |  | 7 (29.2%) | 28 (44.4%) |  | 13 (52.0%) | 34 (48.6%) |  |
| ≤ 5 | 60 (63.8%) | 135 (53.1%) |  | 17 (70.8%) | 35 (55.6%) |  | 12 (48.0%) | 36 (51.4%) |  |
| **CA199** (U/ml)** |  |  | 0.149 |  |  | 0.980 |  |  | 0.499 |
| > 35 | 18 (19.1%) | 33 (13.0%) |  | 3 (12.5%) | 8 (12.7%) |  | 6 (25.0%) | 13 (18.6%) |  |
| ≤ 35 | 76 (80.9%) | 221 (87.0%) |  | 21 (87.5%) | 55 (87.3%) |  | 18 (75.0%) | 57 (81.4%) |  |
| **Tumor size** (cm)* | 4.07±1.39 | 4.15±1.30 | 0.650 | 39.1 ± 13.9 | 40.2 ± 11.6 | 0.745 | 38.9 ± 9.7 | 39.7 ± 10.2 | 0.706 |
| **Distance from anal verge** |  |  | **0.023** |  |  | 0.782 |  |  | 0.480 |
| >5.0cm | 69 (73.4%) | 153 (60.2%) |  | 16 (66.7%) | 40 (63.5%) |  | 16 (57.1%) | 37 (49.3) |  |
| ≤5.0cm | 25 (76.6%) | 101 (39.8%) |  | 8 (33.3%) | 23 (36.5%) |  | 12 (42.9%) | 38 (50.7) |  |
| **Pre-treatment T stage** |  |  | 0.052 |  |  | 0.160 |  |  | 0.534 |
| T1-3 | 76 (80.9%) | 179 (70.5%) |  | 20 (83.3%) | 43 (68.3%) |  | 18 (64.3%) | 53 (70.7%) |  |
| T4 | 18 (19.1%) | 75 (29.5%) |  | 4 (16.7%) | 20 (31.7%) |  | 10 (35.7%) | 22 (29.3%) |  |
| **Pre-treatment N stage** |  |  | 0.230 |  |  | 0.684 |  |  | 0.421 |
| 0 | 24 (6.9%) | 9 (10.3%) |  | 3 (12.5%) | 6 (9.5%) |  | 4 (14.3%) | 16 (21.3%) |  |
| + | 324 (93.1%) | 78 (89.7%) |  | 21 (87.5%) | 57 (90.5%) |  | 24 (85.7%) | 59 (78.7%) |  |
| **Tumor Differentiation**** |  |  | 0.198 |  |  | 0.926 |  |  | 0.123 |
| M/D or W/D | 62 (76.5%) | 209 (82.9%) |  | 17 (89.5%) | 55 (88.7%) |  | 23 (100.0%) | 66 (90.4%) |  |
| P/D or P-M/D | 19 (23.5%) | 43 (17.1%) |  | 2 (10.5%) | 7 (11.3%) |  | 0 (0.0%) | 7(9.6% ) |  |

Data are presented as mean ± standard deviation.

*P* values were calculated using the independent *t*-test or the Mann−Whitney U test for continuous variables, and the chi-squared test for categorical variables. CA199 = carbohydrate antigen 19-9; CEA = carcinoembryonic antigen; M/D = moderately differentiated; NA: not applicable; P/D = poorly differentiated; pCR = pathological complete response; P-M/D: poorly to moderately differentiated; TRG: Tumor Regression grade; W/D: well differentiated.

**Table S5. Predictive performance of artificial intelligence models based on pre-treatment MRI and our model**

| **First author and year** | **Imaging modalities** | **Segmentation**  **method** | **Adding clinical**  **features** | **Training**  **Sample size** | **Validation**  **Sample size** | **Modeling**  **method** | **AUC (95% CI)** | **Accuracy (95% CI)** |
| --- | --- | --- | --- | --- | --- | --- | --- | --- |
| Wu, 2025 (13) | MRI (T2WI) | Manual | None | 401 | 209 and 158,  both external | Deep learning | 0.822 (0.763-0.871)  and 0.786 (0.714-0.847) | 83.7% and 71.9% |
|  |  |  |  |  |  | Sub-regional radiomics | 0.825 (0.766-0.874)  and 0.835 (0.767-0.889) | 70.8% and 83.5% |
| Boldrini, 2022 (12) | MRI (T2WI) | Manual | cT and cN | 161 | 59, external | Radiomics | 0.86 (0.70-1.00) | 0.76 (0.55–0.87) |
| Song, 2022 (11) | MRI (T2WI) | Manual | None | 539 | 135, internal | Radiomics | 0.787 | 92.59% |
| Zhou, 2019 (10) | multiparametric MRI | Manual | None | 318 | 107, internal | Radiomics | 0.773 (0.608-0.937) | 76.6% (68.6%-84.6%) |
| Our model | 3D TRUS | Automatic | None | 348 | 87, internal | Deep learning | 0.92 (0.83 - 0.98) | 86.2% (77.3% - 92.1%) |
|  |  |  |  |  | 103, external |  | 0.86 (0.77 - 0.94) | 83.5% (75.3% - 89.5%) |

AUC = areas under the receiver operating characteristic curve; CI = confidence interval; TRUS = transrectal ultrasound.

**Table S6. AJCC/CAP System for Rectal and Gastroesophageal Cancers**

| **Grading** | **Indication** |
| --- | --- |
| TRG 0 (complete regression) | no residual viable tumor |
| TRG 1 (near-complete regression) | single cells or small clusters of residual tumor cells |
| TRG 2 (partial regression) | residual tumor with evident therapy effect |
| TRG 3 (minimal regression) | extensive residual tumor with little or no therapy effect |

AJCC = American Joint Committee on Cancer; CAP = College of American Pathologists; TRG = Tumor Regression Grade.

**Appendix S1. The** **pre-processing process of 3D transrectal ultrasound data**

The proposed preprocessing workflow comprises the following steps. First, 2D frames are extracted from the original 3D volume at specified sampling intervals (e.g., 6°, 9°, 18°, or a single representative frame). For each extracted 2D frame, the minimum bounding rectangle of the lesion is derived from its predicted contour (output by the semi-supervised segmentation model we developed). The maximum side length (height or width) of this rectangle is recorded as *L*. Subsequently, this rectangle is uniformly expanded by *0.04L* on all sides to define a region of interest (ROI) that includes marginal contextual tissue. The corresponding ROI is then cropped from the original 3D volume. As the cropped region is typically rectangular, symmetric zero-padding is applied along the shorter dimension to obtain a square image patch. Finally, all patches are resized to a standardized resolution of 224×224 pixels to ensure dimensional consistency for model input. This process centers the lesion and normalizes the input format across all samples.

**Appendix S2. Data Availability and Sharing Process**

To facilitate open science while ensuring ethical and secure data use, the de-identified participant data and associated imaging data underlying the findings of this study can be made available to qualified researchers upon reasonable request. The request and provision process is as follows:

**1. Required Application Materials**

Requestors must submit:

(a) a formal research proposal outlining the scientific objectives and analysis plan;

(b) proof of ethical approval from their institutional review board (IRB) or ethics committee for the proposed secondary analysis;

(c) a signed data use agreement that commits to using the data only for the approved purpose, not attempting to re-identify participants, securing the data, and not redistributing it;

(d) a declaration of any potential conflicts of interest.

**2. Review Cycle**

Submitted applications will be reviewed monthly by a designated data access committee comprising the authors and relevant institutional representatives. Applicants can expect an initial response regarding approval or any need for clarification within 8 weeks of submission.

**3. Data Format and Provision**

Approved researchers will receive data in a structured, non-proprietary format. Imaging data will be provided in DICOM format with all protected health information removed. Corresponding annotation data will be provided in CSV format with a comprehensive data dictionary. All data will be shared via a secure, encrypted file transfer platform.
